# Infrapatellar Fat Pad and Knee Osteoarthritis

**DOI:** 10.14336/AD.2019.1116

**Published:** 2020-10-01

**Authors:** Ni Zeng, Zhi-Peng Yan, Xin-Yuan Chen, Guo-Xin Ni

**Affiliations:** ^1^School of Sport Medicine and Rehabilitation, Beijing Sport University, Beijing, China; ^2^Department of Rehabilitation Medicine, The First Affiliated Hospital of Fujian Medical University, Fuzhou, China

**Keywords:** infrapatellar fat pad, knee osteoarthritis, local inflammation, crosstalk

## Abstract

Osteoarthritis is the most prevalent arthritis typically characterized by degradation of cartilage. However, its pathogenesis is not fully understood. Currently, osteoarthritis is best considered a disease of the whole “joint organ”. Infrapatellar fat pad (IFP), an adipose tissue near synovium, is now attaching importance to researchers for its inflammatory phenotype. In this narrative review, a large body of evidence has been gathered for the involvement of IFP in the development of knee osteoarthritis. Additionally, the underlying mechanisms of how IFP can be involved in this process have been proposed. However, further investigations are needed to better understand its precise role in this process and its underlying mechanism, and beyond that, to develop new strategies to slow down the degenerative process and explore an effective and timely diagnosis of the disease.

Osteoarthritis (OA) is the most common joint disease in humans with degenerative joint disease, most often affecting major joints such as the knee, hand, back, and hip [1]. OA is mainly characterized by cartilage damage and osteophyte formation, causing pain and disability [2]. However, its pathogenesis is not fully understood. Currently, OA is best considered a disease of the whole “joint organ.” As local adipose tissue located below the patella, the infrapatellar fat pad (IFP) may interact with other periarticular tissues such as the cartilage, subchondral bone, and synovial membrane, thus playing an important role in the development of knee OA (KOA) [1, 3].

It is evident that the clinical process of OA can be driven by systemic and local inflammation [4-5]. Adipose tissue may produce numerous bioactive substances [6], and consequently induce a low-grade systemic inflammatory state [6-7]. Likewise, IFP may release a variety of inflammatory mediators to induce a local inflammation state. During the past one or two decades, evidence has surfaced for the involvement of IFP in the pathogenesis of KOA. The following review summarizes the latest knowledge about the role of IFP in KOA pathogenesis and proposes underlying mechanisms of how IFP can be involved in the pathogenesis of KOA.

## Anatomy and biologic function of IFP

IFP, also known as Hoffa’s fat pad, was first described by Hoffa in 1904 [3]. Located below the patella, it is an intracapsular but extrasynovial structure [6, 8]. While the IFP is anteriorly limited by the patellar tendon, and posteriorly covered with the synovial lining, it is also superiorly adjacent to the inferior surface of the patella and posteriorly to the intercondylar notch and the cartilage of femoral trochlea [8-9].

The IFP receives a rich vascular supply from its surrounding synovial membrane, and thus aids the healing of the anterior cruciate ligament (ACL) and other nearby structures [10]. The IFP also provides vascular supply to the patellar tendon and inferior patellar pole. Consequently, a year after total knee arthroplasty with excision of the IFP, the patellar tendon shortened considerably [11], which could be attributable to ischemic contracture of the patellar tendon [11-12]. Several joint tissues are involved in the development of OA and OA pain, including the synovial tissue [1, 3, 11], joint capsule [13], and menisci [2, 14-15]. Similar to the synovial membrane, the IFP is richly innervated by nerve fibers containing substance-P, from one posterior articular branch of the tibial nerve [7-8]. Furthermore, in KOA patients, the expression of calcitonin gene-related peptide (CGRP), a vasoactive neuropeptide, in the IFP is higher than that in the synovial membrane and is positively correlated with K-L grade [8]. The IFP is a major source of knee pain in OA patients.

The IFP has several biological roles. Located inside the knee joint, it fills the gaps between joint tissues to stabilize the patella during exercise, thus protecting the knee joint from mechanical damage [13]. During exercise, the pressure and volume of the IFP change throughout the range of motion of the knee joint. At the limits of knee motion, the increased tissue pressure may help to stabilize the patella. The IFP may also act as a cushion between the patellar tendon and anterior tibial plateau [16]. As a result, excision of this fat pad can lead to patella baja and an increased pressure within the patellofemoral joint, which is closely related to anterior impingement and anterior knee pain [11].

Like other adipose tissues, the IFP is capable of secreting various cytokines and adipokines and therefore involved in the development of joint disease [14]. More will be discussed below as to their biological functions and how they participate in the local inflammation state. Moreover, a number of studies have indicated that the IFP could act as a reservoir of reparative cells for tissue engineering applications [18]. For instance, adipose-derived mesenchymal stem cells (MSCs) possess a much greater chondrogenic potential than MSCs derived from other tissues to treat the inflammatory aspect of OA [18-19]. More importantly, after comparing the implications for cartilage repair of different connective tissue progenitors (MSCs derived from IFP, synovium, and periosteum), Mantripragada et al. found that, although the connective tissue progenitors were available in all of these three tissues, IFP is a preferred tissues source in almost all patients [20].

## Involvement of IFP in KOA pathogenesis

Evidence indicates that IFP exhibits unique pathological manifestations in KOA patients [1, 21]. When compared with their healthy counterparts, the IFP of KOA patients presented an increase in inflammatory infiltration, vascularization, and thickness of the interlobular septa. In addition, a more crown-like structure and considerably higher VEGF, MCP-1, and IL-6 proteins were observed [1, 22]. In parallel, compared with the subcutaneous adipose tissue (SAT) within the same KOA patient, the IFP exhibited an increase in fibrosis and vascularization, more lymphocytic infiltrating interlobular septum, and smaller fat lobules [1, 21-23]. These findings indicate that the IFP may play a deleterious role in KOA by affecting joint homeostasis due to its in?ammatory phenotype.

Of all these pathological manifestations, fibrosis may be the most typical characteristic of the IFP in OA patients [1]. Histological analyses indicate that the extent of fibrosis in the IFP was higher than that in the SAT [24]. Histological changes, such as IFP fibrosis, may not only be involved in molecular OA processes by changing cytokines production [1, 23], but they may also alter the IFP biomechanical properties, thus modifying its ability to absorb gravitational forces exerting on the knee joint, and promoting and perpetuating joint damage [1].

As recognized, fibrosis could be induced by mechanical stress [23, 25]. While fibrosis seems to increase with obesity in the study of mice, it is not associated with obesity in the study of humans [25]. Discordance in these results could be attributable to the different responses to mechanical loading or stress between humans and mice that result in maximal fibrosis in human IFP tissue. Opposite to the mouse IFP, the human IFP cannot expand in volume with obesity [25], which could contribute to increased mechanical stress and subsequent fibrosis in human IFP tissue. Additionally, fibrosis may aid repair in the process of tissue healing after injury [3]. Such effect could provide an explanation to not only the increased degree of IFP fibrosis in patients with advanced OA and in high fat feeding animals, but also the correlation in the increase in IFP fibrosis and the degree of joint damage [23, 25].

More direct evidence for the involvement of IFP in KOA pathogenesis comes from magnetic resonance imaging (MRI), a reliable means of providing quantitative information of IFP, including its volume, maximum area, and signal intensity [26, 27]. It was found that IFP volume in OA patients is larger than that in their healthy counterparts [28] and increases with age [2, 29]. More importantly, the correlation between these MRI features and cytokine level, joint structure, and clinical syndromes has been extensively investigated in KOA patients. Firstly, a correlation has been found between the MRI features and serum levels of cytokines. For example, Wu et al. [30] found that serum level of ghrelin was associated with IFP signal intensity alteration, and another study demonstrated that serum IL-17 and resistin were associated with reduced IFP volume and/or increased abnormal signal intensity alteration, whereas serum adiponectin had opposite associations [31]. Secondly, IFP volume in OA patients was correlated with X-ray K-L grades [25, 29]. Likewise, IFP signal intensity in OA patients was significantly correlated with knee structural abnormalities (such as knee cartilage volume, cartilage defects, and bone marrow lesions (BMLs), the occurrence of knee replacement and radiographic progression, respectively [32-33]. Thirdly, a large body of evidence indicates that MRI features of IFP may be associated with clinical symptoms in KOA patients [2, 34], as summarized in Table 1.

**Table 1:** The relationship between MRI features of IFP and knee characteristics.

Ref. (year)	Subjects	Findings
33 (2018)	KOA patients	Increased signal intensity in the IFP was associated with knee structural abnormalities in tibiofemoral compartment.
34 (2018)	KOA patients	IFP signal intensity is associated with the occurrence of knee replacement.
28 (2017)	KOA patients	1.The increase in 3D MRI heterogeneity was greater in progressor and OA knees than non-progressor knees and healthy knees, respectively. 2. Increase in 3D IFP MRI signal and signal heterogeneity may be associated with radiographic/symptomatic progression of OA, when compared to non-progressive OA or healthy knees
35 (2016)	Older adults	The IFP signal abnormalities has a potentially important role in OA progression.
81 (2015)	Adults without KOA	1.IFP at baseline was associated with reduced knee pain at follow-up and lateral tibial cartilage volume loss; 2. IFP size is not simply a marker of systemic obesity.
82 (2015)	PFJ OA patients	1. IFP volume was greater in the PFJ OA group than controls and it was directly related to PFJ OA pain; 2. Larger IFP was associated with worse pain.
2 (2015)	Older adults	1.IFP maximal area in women was significantly associated with changes in knee pain and reduced loss of medial and lateral tibial cartilage volume; 2. IFP plays a protective role in joint degeneration in the elderly.
36 (2014)	Older adults	1.IFP maximum area was significantly associated with joint space narrowing and medial osteophytes, knee tibial and patellar cartilage volume, tibial cartilage defects, any BMLs, and knee pain on a flat surface; 2.IFP maximum area is beneficially associated with radiographic OA, MRI structural pathology and knee pain on a flat surface suggesting a protective role for IFP possibly through shock absorption.
15 (2014)	KOA patients	1.The severity of inflammation in the IFP were associated with the severity of pain in KOA; 2.DCE-MRI is a promising method to study the impact of inflammation in KOA
30 (2010)	KOA patients	Subjects who are prone to growth or enlargement of the IFP may also be more prone to symptomatic OA.

KOA, knee osteoarthritis; OA, osteoarthritis; PFJ, Patellofemoral joint, DCE-MRI, dynamic contrast-enhanced magnetic resonance imaging; BML, body mass index; IFP, infrapatellar fat pad

Pain is a common clinical symptom among KOA patients. Those with increased IFP volume tend to have symptomatic OA [2, 29]. In parallel, IFP signal intensity alternation was positively associated with the increase in knee pain [34]. IFP maximal area at baseline was associated with reduced knee pain [2]. Notably, IFP inflammation was associated not only with pain, but also with all subscales of the Knee injury and Osteoarthritis Outcome Score (KOOS), showing that increased IFP inflammatory manifestations relate to more severe knee-related symptoms and impaired function in daily living, knee-related quality of life, and function in sport and recreation [15]. By contrast, a recent study claimed that IFP size does not appear to be associated with knee pain [34]. This study used a between-person matched pair and a within-person between-knee design to enhance the external validity of its findings and reduce the influence of environmental, social, and psychological factors. However, even if no association is observed between the imaging parameters of IFP and knee pain, it does not mean that no association exists between IFP and knee pain. IFP may drive pain via biochemical interactions that stimulate nociceptive fibers, even if the biochemical status may not alter the overall MRI signal [34].

It is worth noticing that IFP features can be used to predict OA development and progression [2, 35], thus providing additional evidence for the involvement of IFP in KOA pathogenesis. A study focusing on the elderly before the occurrence of KOA found that the change in IFP signal intensity related to the abnormal structure of the knee joint and clinical symptoms cross-sectionally and longitudinally, suggesting that IFP signal intensity is an important imaging marker of KOA [35]. Further, the maximum area of the IFP relates to the annual changes in the thickness of tibial cartilage, indicating that the IFP has a positive protective effect on knee pain symptoms and cartilage damage in elderly women [35].

## Possible mechanisms of IFP in KOA pathogenesis

Although accumulating evidence demonstrates the involvement of the IFP in OA pathogenesis, its precise mechanism remains unclear. It is supposed that the IFP may be involved in the local inflammation state and/or that it might interact with other periarticular tissues, thus contributing to the development of KOA. Certainly, many factors such as obesity, bone fracture, and aging are related to abnormal loading or abnormal cartilage, and eventually lead to cartilage injury. During this process, the released cartilage-specific autoantigens may activate the immune response of the IFP. Activated immune cells subsequently interact with adipocytes and secrete a variety of cytokines and adipokines to participate in the local inflammation state. The involvement of the IFP in the development of OA may be associated with the potential mechanisms described in the following section.

## Inflammatory Mechanisms of IFP in KOA

### Immunophenotype of immune cells

The immune response is activated following the damage of cartilage. The role of immune responses has been demonstrated in the pathogenesis of OA [24], during which both innate and adaptative immunity may play important roles [36].

As innate immune mediators, macrophages contribute to the development of KOA by releasing products. Different types of macrophages release different inflammatory products. For example, M1 macrophage is an important immune cell for host defense and may transmit an inflammatory response by producing cytokines such as IL-1β, TNF- α, IL-6 and IL-12 [6]. By contrast, M2 macrophage expresses mannose receptor CD206 and a scavenger receptor, and it mainly produces IL-10, but not IL-12 and IL-23, indicating that it is an anti-inflammatory cell [6, 37].

Many double-positive cells were observed in the IFP [38], underlining that the division between M1 and M2 macrophages is a gliding scale [37]. Of note, macrophages in the IFP may display a mixed pro- and anti-inflammatory phenotype [13, 39], and M2-type macrophages may interact with adipocytes or other cells to release the inflammatory products [37]. Accumulating evidence indicates that CD206+ cells are involved in the inhibition of catabolic processes. For example, significantly more CD206+ macrophages were present in the IFP of OA patients compared with the SAT of the same OA patients and the IFP of ACL injury patients, and IFP derived fat-conditioned media inhibited catabolic processes in end-stage OA cartilage [37]. However, M2-type macrophage is regarded as a profibrotic cell during wound healing [23,37]. The increased frequency of CD14+CD206+ M2-type macrophages in the IFP deposits of obese patients with OA correlate significantly with the fibrosis of the IFP, implying that the IFP may not play a protective role in the development of OA. Moreover, the transcription of several PPARγ-regulated genes is upregulated in the M2 macrophage, including encoding arginase 1, macrophage mannose receptor 1, and IL-1 receptor antagonist. PPARγ, which is a regulator of the glucose and lipids metabolism, has an anti-inflammatory role in OA cartilage [1, 3, 40].

Along with macrophages, many other immune cells present in the IFP may also contribute to the development of OA, including the adaptive compartment (T cells and B cells). Klein-Wieringa et al. [39] used flow cytometry to analyze the proportion of immune cells in OA patients and found a higher ratio of mast cell ratio and lower proportion of T cells in the IFP compared with SAT. In addition, similar to that of the synovium, the immune cells in the IFP showed an active, proinflammatory phenotype [39].

In KOA patients, the proportion of CD4+ T cells in the IFP is higher than that of CD8+ T cells, however, CD8+ T cells present a higher level of activation. There are more CD8+ T cells in IFP in OA patients with higher radiographic grading [24]. In addition, the CD8+ T cells in the peripheral blood appear to be active, but whether the activation of the CD8+ T cells in IFP is recruited into the tissues by preactivated circulating peripheral blood CD8+ T cells remains unclear [24]. An animal study suggested that CD8+T cells may modulate joint angiogenesis and matrix turnover, and thus contribute to the development of OA by up-regulating TIMP-1 [41]. However, further investigations are warranted to understand the underlying mechanisms of how adaptive immune cells, especially highly activated CD8+ T cells in the IFP, are involved in the pathogenesis of OA.

As discussed above, both innate and adaptative immunity contribute to cartilage injury; they interconnect in the process through some cytokines. Subsets of T cells involved in the regulation of macrophage phenotype. For example, CD4+ regulatory T cells in the adipose tissue of lean mice are more abundant and have an anti-inflammatory protective effect by inhibiting inflammatory macrophages [40]. In parallel, in the helper cell of CD4+ T cells, Th1 secrets various cytokines, such as IFN γ, to induce macrophages polarized into M1 type macrophages and produce pro-inflammatory cytokines (for example, TNF and IL-6) [3, 40], whereas Th2 type cytokines, such as IL-4, would induce macrophages to become M2 type and produce anti-inflammatory cytokines (for example, IL-10) [40]. CD8+ effector T cells and Th1 cell related factors can initiate the recruitment and activation of macrophages in adipose tissue and promote the inflammatory cascade reaction of insulin resistance. Therefore, the disorder of the balance between type Th1 and Th2 type signals caused by obesity may affect the recruitment and activation of macrophages in the adipose tissue, resulting in pathogenic and inflammatory conditions or non-inflammatory and protective environments [40].

### Secretory profile of IFP

In addition to acting as a source of energy, adipose tissue can affect other tissues by secreting many cytokines and adipokines [6, 14]. In this regard, the IFP could interact with other joint tissues by secreting these factors to influence the progression of OA through its biological function [13, 40].

*Cytokine:*Immune cells in the IFP secrete a variety of cytokines, including pro-inflammatory and anti-inflammatory cytokines. In KOA patients, the expression of IL-6 and its receptor sIL-6 in the IFP was found to be much higher than that in the subcutaneous tissue, indicating that IL-6 may affect other joint components through paracrine, and play a crucial role in the development of OA [1, 6]. Furthermore, when compared with control patients, the IFP exhibited a higher expression of VEGF in OA patients, which was positively associated with the vascularization degree of synovium, suggesting an interaction between the IFP and synovium [1].

By contrast, inflammatory cytokines such as TNF-α, IL-4, IL-10, PGE2, and IL-1β may also contribute to the development of joint disease; however, their productions in the IFP in OA patients are similar to those in the subcutaneous fat in OA patients and the IFP in control subjects [14, 40]. Studies on these cytokines are exhaustive because of their important roles in inflammation. For example, IL-1β has been used to induce normal chondrocytes in the phenotype of OA chondrocytes in vitro [42], and the role of anti TNF-α targeted drugs has also been suggested as an option for treating arthritis [43].

*Adipokine:*In addition to cytokines, the IFP can secrete adipokines via adipocytes, thus contributing to the inflammation of knee joint. The so-called “classical” adipokine, primarily expressed by adipocytes, includes leptin, adiponectin, and resistin [3, 40]. Some newly discovered adipokines, whose roles in OA have been gradually revealed, include FABP4 (fatty acid binding protein), WISP2 (WNT1 inducible signaling pathway protein 2), and Chemerin [44-46].

Adipokines may differ with their presence in the IFP and their role in OA. Many adipokines are expressed in the IFP of KOA patients. Compared with the SAT in OA patients, the IFP exhibited significantly higher expression in adiponectin, visfatin, and chemerin, and lower expression in leptin [6, 47]. Nevertheless, compared with healthy individuals, the IFP in OA patients exhibited significantly higher expression in leptin, chemerin, FABP-4, and WISP2, and no significant difference in visfatin [44, 46, 47]. In addition, some adipokines that are involved in the development of OA, such as SAA3 (serum amyloid A3) and Lipocalin2 [48, 49], are not found to be expressed in IFP. A few others, such as SFRP5(Secreted frizzled-related protein-5) and mi-RNA in exosome, have not been proven for their presence in the IFP and their role in OA [50, 51].

Leptin is a central regulator in energy homeostasis [52]. Evidence indicates that leptin is involved in the development of OA [23]. Firstly, high circulating leptin level in obese individuals may protect cartilage from osteoarthritic degeneration, and intra-articular injection of leptin can stimulate the formation of proteoglycans and growth factors [1]. Besides, leptin induces chondrocyte anabolism through regulating the expression of MMP-2, MMP-9, ADAMTS-4/5, NO, PGE2, IL-6, IL-8, etc [17]. For osteoblasts, leptin produced by IFP can also regulate the synthesis of ALP, TGF-β, osteocalcin and type I collagen in osteoblasts [1, 3]. Furthermore, it can affect the process of inflammatory reaction by affecting IL-1β and affecting the activation of mast cells and Th1 cells [1].

Resistin is mainly produced by monocytes and macrophages of adults. It may induce macrophages to secrete IL-6, IL-12 and TNF- α, and lead to the loss of proteoglycans in cartilage [53]. Its concentrations in serum and synovial fluid are associated with clinical severity in knee OA patients [54]. In a mouse study, the intra-articular injection of resistin can induce arthritis, suggesting that resistin is a pro-inflammatory cytokine [55].

Different from leptin and resistin, adiponectin is an anti-inflammatory cytokine. It can promote insulin sensitivity and protect ischemia and reperfusion injury of cardiovascular tissue [56]. Unlike in cardiovascular disease, adiponectin may act as an inflammatory mediator of joint disease [56]. Plasma adiponectin level is positively correlated with plasma COMP and MMP-3 levels, as well as clinical symptoms and imaging severity of KOA [31]. In parallel, in vitro experiments found that adiponectin increases nitric oxide and MMP production in human osteoarthritic chondrocytes, suggesting that adiponectin may be involved in the destruction of cartilage matrix [57].

*Fatty acids and fatty acid derivatives.*Adipose tissue also secretes fatty acids and fatty acid derivatives, which are highly immune-regulated and can modulate the phenotype of macrophages and CD4+T cells [3, 58]. Among them, 14 fatty acids and fatty acid derivatives have been found to be associated with OA, especially the anti-inflammatory lipid mediator lipoxin A4, thromboxane B2, and arachidonic acid [59]. With regard to arachidonic acid, higher secretion of arachidonic acid (20:4n-6 polyunsaturated FA) was found in the IFP of OA patients compared to their healthy counterparts. It may lead to the inflammation of cartilage via increasing PGE2 production. On the other hand, the mobilization of 22:6n-3 polyunsaturated fatty acid (docosahexaenoic acid) from the IFP was higher in OA patients than non-OA patients. N-3 polyunsaturated fatty acid can protect cartilage joint tissues by reducing the production of COX-2, pro-inflammatory cytokines, and cartilage-degrading enzymes [60]. In this regard, IFP may induce both protective and disease-aggravating activities in the KOA. More studies are required to understand the role of fatty acids and fatty acid derivatives derived from the IFP in KOA patients.

**Figure 1.**
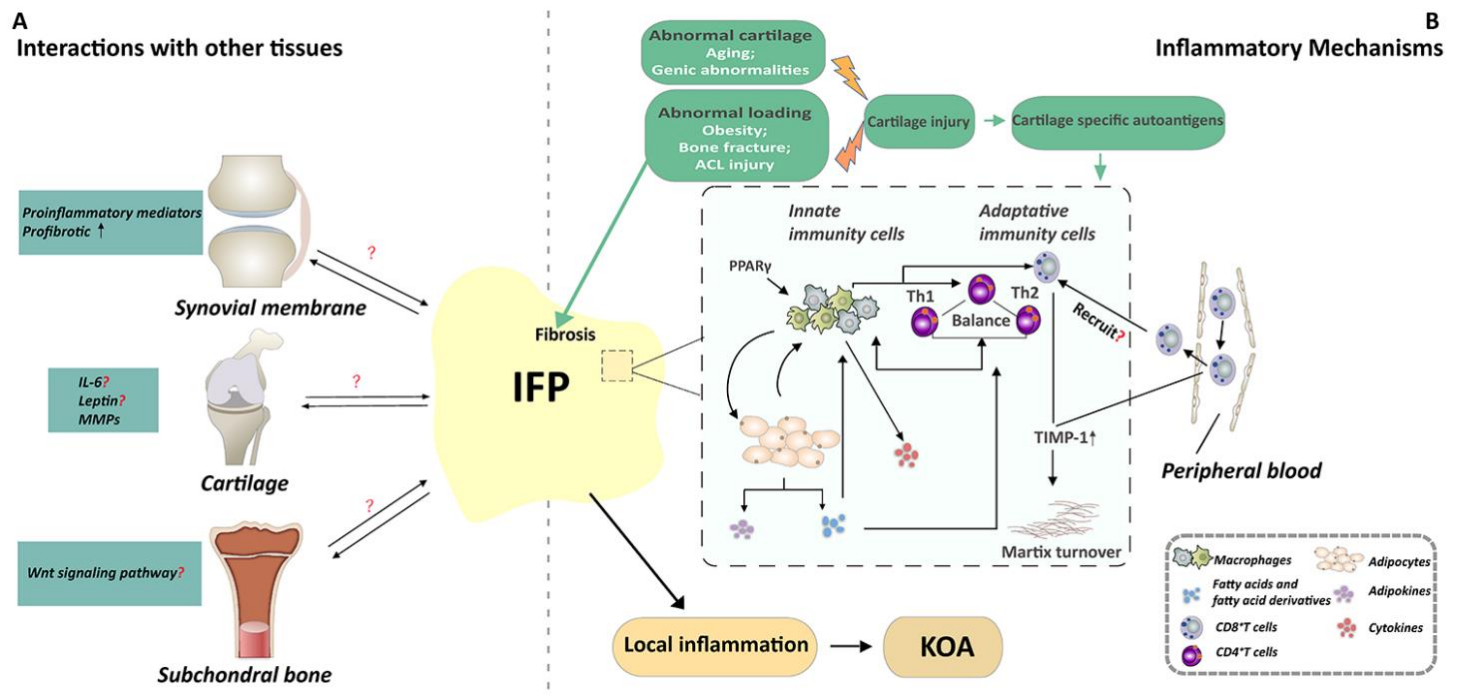
A mechanistic model depicting how IFP is involved in OA pathogenesis. It is supposed that the IFP may be involved in the local inflammation state and/or that it might interact with other periarticular tissues, thus contributing to the development of KOA. (A). IFP may interact with cartilage, synovial membrane and subchondral bone. IFP may interact with the synovial tissue through affecting the inflammatory response and promoting the fibrosis of the synovial cells. In parallel, IFP plays either a deleterious or a protective role on cartilage by altering the expression of IL-6 or leptin or MMPs. While the influence of IFP on subchondral bone has not yet been investigated clear, IFP may interact with subchondral bone through Wnt signaling pathway. Reciprocally, these joint tissues could also modulate the IFP. (B). Many factors such as obesity, bone fracture, and aging are related to abnormal loading or abnormal cartilage, and eventually lead to cartilage injury. Then, the immune response of IFP may be activated by the cartilage specific autoantigens. The immunophenotype of immune cells of IFP is related to inflammatory phenotype of IFP, which is the potential mechanisms of the involvement of IFP in the development of OA. Macrophages consist of is type M1 and M2 macrophage. PPARγ-regulated genes are upregulated in M2 macrophage. Macrophages can interact with T cells and adipocytes to participate in local inflammation state. First, CD4 ?T cell also called Th cells, it contains two subtypes named Th1 and Th2. The balance between type Th1 and Th2 type signals may affect the recruitment and activation of macrophages in the adipose tissue, resulting in pathogenic and inflammatory conditions or non-inflammatory and protective environments. Then, adipocytes secrete fatty acids and fatty acid derivatives which are highly immune-regulated, to modulate the phenotype of macrophages and CD4+T cells. Moreover, CD8+ T present a higher level of activation in the IFP, and the activated CD8+ T cells in IFP may recruited into the tissues by preactivated circulating peripheral blood. In addition, CD8+T cells may modulate matrix turnover, and thus contribute to the development of OA by up-regulating TIMP-1. These activated immune cells and adipocytes alter the inflammatory phenotype of IFP, participate in the local inflammation of the whole joint, and ultimately lead to KOA. Short title of the first figure. The figure caption should begin with a title (an overall descriptive statement of the figure) followed by additional text. The legends should be placed immediately after each figure.

## Interactions with other tissues

### Interaction with cartilage

Evidence indicates that the IFP may interact with cartilage. On the one hand, the interaction of the IFP has been extensively investigated with cartilage under various conditions. For example, the behaviors of bovine chondrocytes were examined after being cultured with IFP derived FCM (fat-conditioned media) from OA patients, and the results suggested that the IFP has either a deleterious [61] or a protective [37] effect on cartilage. The discrepancy in the types of effect observed may be attributable to different experimental designs and the OA stage of the patients. Additional experiments are required to investigate the effect of FCM on the entire osteoarthritic process and to examine the human cartilage in more detail [36]. In addition, the interaction between healthy/traumatized IFP and healthy/traumatized cartilage was examined. The findings indicated that human IFP-derived medium enhanced degenerative changes in the traumatized bovine cartilage, but not in the healthy cartilage [62]. Another study used a tissue explant co-culture model (not IFP-derived medium) to assess the effect of healthy IFP tissue on cartilage and found that the healthy IFP did not promote the degradation of cartilage [63]. It appears that the interaction of the IFP with the cartilage depends on the condition of both tissues. In turn, the effect of traumatized cartilage on the IFP was studied by treating IFP-derived adipocytes and IFP adipose-derived stromal cells (ADSC) with traumatized cartilage-conditioned medium (TC-CM) followed by an analysis of cytokine expression [62]. The results suggest that IFP aggravates post-traumatized cartilage degeneration, and IL-6 may mediate the interaction between these two tissues. Notably, several IFP secreted cytokines and adipokines may induce cartilage change. For example, leptin can affect the generation of MMPs in cartilage [61]. Further investigations are needed to understand the role of these factors in the cross-talk between the IFP and cartilage.

Briefly, there are four types of crosstalk between the IFP and cartilage: healthy IFP “talks” to healthy cartilage, abnormal IFP “talks” to abnormal cartilage (i.e., traumatized cartilage, cartilage of arthritis patients), healthy cartilage “talks” to abnormal IFP, and abnormal cartilage “talks” to abnormal IFP. Distinguishing between the “speaker” and the “listener” is important for identifying the protective or degenerative role of the two tissues in the process of disease progression. Currently, it remains unknown as to how healthy IFP affects healthy or abnormal cartilage; however, knowing this may help us to explore the underlying mechanisms of IFP in KOA pathogenesis.

### Interaction with the subchondral bone

The subchondral bone plays a crucial role in the pathogenesis of OA. Currently, our understanding is still limited as to whether and how the IFP and subchondral bone interact. An in vitro study demonstrated that adiponectin has an effect on the osteoblasts derived from osteophytes, through the p38 MAPK signaling pathway, rather than the Wnt signaling pathway [64]. This finding contradicts our expectation somewhat since the Wnt signaling pathway has a role in the pathological process of OA [65, 66] and, more importantly, several adipokines activate the Wnt signaling pathway, including leptin [67] adiponectin [68], SFRP5, and WISP2 [13,69]. It is likely that exogenous adipokines fail to activate the Wnt signaling pathway derived from osteophyte osteoblasts, and to participate in the formation of osteophyte. Nevertheless, some other adipokines involved in the development of OA are supposed to participate in IFP-subchondral bone interaction. For example, osteoblasts were sensitive to visfatin by inducing the expression and production of the proinflammatory cytokines (such as IL-6 and MCP-1) [70]. Likewise, SAA3 has an important role in extracellular matrix repair, bone remodeling, bone resorption, and skeletal development [71, 72]. In the future, more evidence is required to understand whether and how the IFP and subchondral bone may interact.

### Interaction with synovium

Unlike cartilage and the subchondral bone, synovium shares a similar immune phenotype and pathological change to the IFP during OA development, strongly implying an interaction between them [73, 74]. In an in vitro study, human OA fibroblast-like synoviocytes were cultured in FCM made from either IFP or subcutaneous fat in KOA patients, and the results suggested that the IFP may interact with the synovial tissue through affecting the inflammatory response and promoting the fibrosis of the synovial cells [75]. Actually, there exists a two-way communication between the IFP and the synovium. Some pro- or anti-inflammatory cytokines like TNFα, IL-1β, or IFNγ, known to be secreted by synovium can induce the inflammatory response of IFP. On the other hand, it was demonstrated that, after intraarticular injection of monoiodoacetate in mice, the necrosis of adipocytes and fibrosis of the IFP are associated with synovial hyperplasia [75-77]. Further studies are needed to explore the adipokines and signaling pathway involved in the interaction.

## Conclusions and future directions

In this review, up-to-date information has been gathered to analyze the role of IFP in KOA pathogenesis, and to propose the underlying mechanisms of how IFP can be involved in this process. In recent years, IFP has drawn increasing attention due to its important role in OA pathogenesis. However, our current understanding is still limited. For instance, its precise role (protective or promoting role) remains unclear. More investigations are needed to better understand its precise role in this process and its underlying mechanism, and beyond that, to develop new strategies to slow down the degenerative process and explore an effective and timely diagnosis of the disease.

MRI has been extensively used to obtain quantitative information of the IFP in OA patients, thus providing direct evidence for involvement of the IFP in KOA pathogenesis. Nevertheless, the following concerns need to be addressed in the future: (1) the correlation between MRI features and pathological changes of the IFP; (2) the mechanisms behind the role of the IFP in OA symptoms and cartilage defects; (3) the relationship between the serum level of some new adipokines and MRI features of the IFP; (4) how MRI features change with OA symptoms after interventions, such as anti-inflammatory drugs; (5) whether MRI features of the IFP can be used as an imaging biomarker of OA; and (6) whether local adipose tissues in other joints are related to joint pain.

It remains a big concern as to whether the IFP should be retained during TKA. A significant association was revealed between the IFP maximal area and the reduced loss of cartilage volume, indicating that the IFP has a protective effect on cartilage. Nevertheless, compared with TKA patients with IFP retention, those with IFP resection present similar knee function [11]. Although less anterior knee pain was also suggested in patients with IFP resection, the incidence of knee pain following TKA is generally quite low [78]. Additional benefits for IFP retention may include the reduction in wound complications, such as delayed wound healing and persistent wound drainage [79]. In the future, randomized controlled trials (RCTs) are needed to determine whether the IFP should be retained or resected during the TKA procedure.

The IFP may secrete a variety of cytokines and adipokines to participate in the local inflammation of the knee joint, thus contributing to the development of KOA. While more and more adipokines are being found, such as Lipocalin2, SFRP5, and mi-RNA, their roles in the development of OA are rarely known [51, 59, 74]. Understanding the complexity of the adipokines network in the IFP and their interactions with other joint components is essential for explaining the pathogenesis of OA, thus developing new treatment strategies and interventions in inflammatory joint disorders. Additionally, it is proposed that the interaction between IFP and other periarticular tissues should contribute to the development of OA. Further investigations are warranted to better understand whether and how IFP interacts with other periarticular tissues, how IFP may change in the process of OA, and which tissue (IFP and other periarticular tissues) may initiate the development of OA.

As discussed earlier, stem cell therapy may be a promising option for cartilage repair using IFP-derived MSCs. Nevertheless, a number of concerns should be addressed. For example, increasing evidence suggests that the cell population obtained by culture expansion is not homogeneous to MSC cell population [20]. As such, reproducible therapeutic efficiency could be compromised by this cell population manufactured. Clonal selection and isolation of the heterogeneous cell population from the connective tissues is regarded as an optimizing means to culture expanded cells harvested from patients [20, 80]. Notably, the role of MSCs in the cartilage regeneration is attributable to the paracrine activity of exosomes. MSCIFP-derived exosomes could protect cartilage regeneration, inhibit the chondrocyte apoptosis and balance the anabolic and catabolic processes [20]. In addition, MSCs-derived exosomes are more stable than MSCs themselves and have immune privileged to a certain degree. Moreover, it is easier to acquire MSCs-derived exosomes than to select and isolate the heterogeneous cell population [80]. Therefore, MSCIFP-derived exosomes could be a novel potential therapeutic strategy for OA, which may be superior to the stem cell therapy in many ways. Further investigations should focus on the safety and efficacy of MSCIFP-derived exosomes in cartilage repair.

Finally, accumulating evidence indicates the involvement of IFP in the development of KOA. IFP is a local adipose tissue located below the patella. There are many other similar adipose tissues intra-articular and around the knee joint. An interesting concern is therefore raised as to whether they may function similarly to the IFP, or even act together with the IFP in the development of OA. More investigations are needed to address this concern.
